# Draft genome sequence of *Bacillus velezensis* strain 3TSA-3, a potential probiotic for Pacific white shrimp *Penaeus vannamei* postlarvae isolated from commercial hatchery tanks

**DOI:** 10.1128/mra.01208-23

**Published:** 2024-03-19

**Authors:** Guillermo Reyes, Irma Betancourt, Martha Borbor, Bonny Bayot

**Affiliations:** 1 Centro Nacional de Acuicultura e Investigaciones Marinas, CENAIM, Escuela Superior Politécnica del Litoral, ESPOL, Guayaquil, Ecuador; 2 Facultad de Ingeniería Marítima y Ciencias del Mar, FIMCM, Escuela Superior Politécnica del Litoral, ESPOL, Guayaquil, Ecuador; University of Maryland School of Medicine, Baltimore, Maryland, USA

**Keywords:** *Penaeus vannamei*, probiotics, aquaculture, vibriosis, hatchery

## Abstract

We report the draft genome of *Bacillus velezensis* strain 3TSA-3, isolated from Pacific white shrimp *Penaeus vannamei* postlarvae collected from a hatchery tank with high survival despite the presence of pathogenic *Vibrio*. The strain possesses genes encoding bacteriocins and lacks virulence factor genes, characteristics for a potential aquaculture probiotic.

## ANNOUNCEMENT

*Bacillus velezensis* is a probiotic bacterium beneficial to adult stages of *Penaeus* (*Litopenaeus*) *vannamei* (1–3). *B. velezensis* strain 3TSA-3 was isolated from *P. vannamei* postlarvae collected from a commercial larviculture tank in Santa Elena province, Ecuador, on 9 March 2021. Despite being affected by vibriosis, the postlarvae exhibited high survival rates at harvest. The strain 3TSA-3 was isolated from shrimp postlarvae macerated in tryptone-casein soy broth (TSB) with 2% sodium chloride. The mixture was then cultured on tryptone-casein soy agar (TSA). After 24 h of incubation at 30°C, colonies suggestive of *Bacillus* were subcultured on TSA.

Genomic DNA (gDNA) of *B. velezensis* 3TSA-3 was extracted from an overnight culture on TSA using a DNeasy UltraClean Microbial Kit (Qiagen, The Netherlands). The quality and concentration of gDNA were assessed using a Denovix DS-11 spectrophotometer (Denovix Inc., USA).

**Fig 1:**
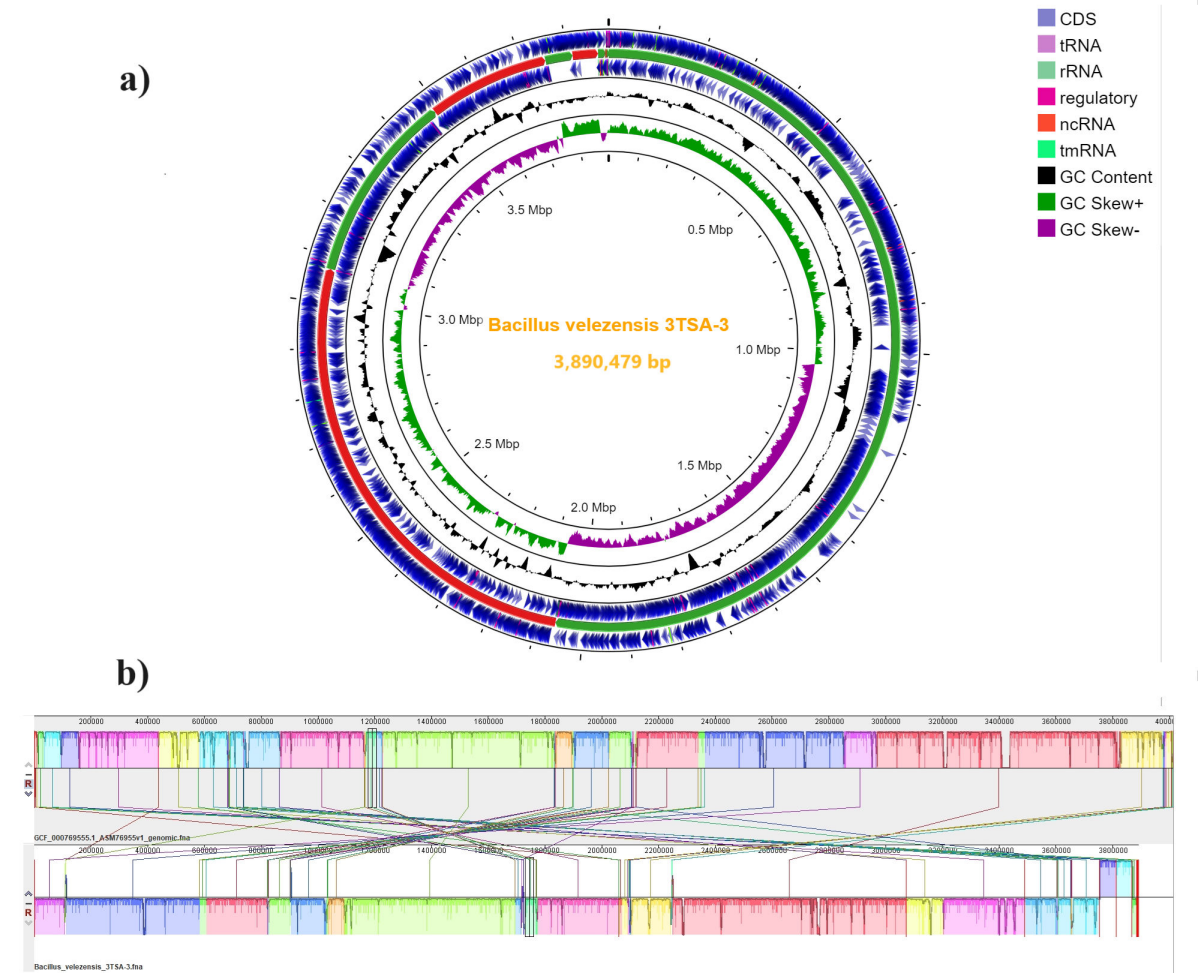
Circular genome map and Mauve alignment of the *Bacillus velezensis* strain 3TSA-3. (a) Multiple scaffolds (13) of the incomplete genome of 3TSA-3 showing DNA coding sequences (CDSs), tRNAs, rRNAs, regulatory, ncRNA, tmRNA, GC content, and GC skews. (b) Mauve alignment of the 3TSA-3 draft genome with the *B. velezensis* reference genome strain JS25R (Genbank accession number GCA_000769555.1).

The whole-genome library was prepared using the NEBNext ULtra DNA Prep Kit for Illumina (350 bp), and sequencing was performed by Novogene Inc. (Sacramento, USA) using the Illumina NovaSeq 6000 PE150. Whole-genome sequencing analyses were performed using the Bactopia pipeline v3.0.0 (4) with default software and parameters. The quality of the raw reads was assessed using FastQC v0.12.1. This analysis yielded a total of 8,254,750 high-quality reads with a Phred score of 36.5. *De novo* assembly was performed with Shovill v1.1.0. A circular genomic map (Fig. 1a) was generated using Proksee server v1.1.0 (5). 3TSA-3 was identified as *B. velezensis* based on the Kraken v2.0 taxonomic classification system (6) and 16S rRNA gene sequence homology using BLASTN (7). The contigs were ordered and oriented on one reference genome of *B. velezensis* (Strain JS25R, Genbank accession number GCA_000769555.1) using CSAR v1.1.1 (8). Average nucleotide identities with the reference genome *B. velezensis* strain JS25R were calculated using fastANI v1.3.3 (5), which showed a close similarity (98.05%) of strain 3TSA-3 strain to *B. velezensis* strain JS25R. In addition, the contigs of the circular genome were aligned to the reference genome using Mauve v20150226 (9) (Fig. 1b). Genome annotation was performed using the NCBI Prokaryotic Genome Annotation Pipeline (PGAP) (10). Quality and statistics of the assembled genome sequence were performed using QUAST v5.2 (11). The assembly data revealed a total of 3,890,479 bp in 13 scaffolds of >500 bp (minimum size of 630 bp, maximum size of 2,060,921 bp, and N50 contig length of 2,060,921), with a GC content of 46.43% and an average read coverage of 312×. Homology-based gene prediction identified a total of 3,882 genes, including 3,684 protein-coding sequences (CDSs), 98 RNA genes (82 tRNA, 11 rRNA, and 5 ncRNA), and 100 pseudogenes. Several genes encoding enzymes involved in sporulation, biosynthesis of siderophores and arsenate bioremediation, biosynthesis of indole acetic acid, and biosynthesis of bacteriocins, such as the uberolysin/carnocyclin family, were detected.

The discovery of effective probiotics and their genomic data are fundamental to the sustainability of the shrimp farming industry, especially considering the global threat of multi-resistance to antibiotics, which dictates the need to explore natural cost-effective alternatives such as probiotics.

## Data Availability

This whole-genome shotgun project of *B. velezensis* 3TSA-3 has been deposited in GenBank under the accession number JAXGGL000000000. The raw sequence reads are available under the SRA accession number SRX22663519, and Bioproject accession number PRJNA1046100.
